# Unveiling purine metabolism dysregulation orchestrated immunosuppression in advanced pancreatic cancer and concentrating on the central role of NT5E

**DOI:** 10.3389/fimmu.2025.1569088

**Published:** 2025-04-01

**Authors:** Junqian Zhang, Xiaobo Zhang, Ruixin Wu, Chang-Sheng Dong

**Affiliations:** ^1^ Henan Key Laboratory of Cancer Epigenetics; Cancer Institute, The First Affiliated Hospital, and College of Clinical Medicine of Henan University of Science and Technology, Luoyang, China; ^2^ Department of Hepatobiliary Surgery, The First Affiliated Hospital, and College of Clinical Medicine of Henan University of Science and Technology, Luoyang, China; ^3^ Preclinical Department, Shanghai Municipal Hospital of Traditional Chinese Medicine, Shanghai University of Traditional Chinese Medicine, Shanghai, China; ^4^ Cancer Institute of Traditional Chinese Medicine/Department of Oncology, Longhua Hospital, Shanghai University of Traditional Chinese Medicine, Shanghai, China

**Keywords:** purine metabolism, immunotherapy, CAFs, NT5E, pancreatic cancer

## Abstract

**Background:**

The dismal efficacy of immunotherapy for Pancreatic cancer (PC) can be predominantly ascribed to its distinctive cold-tumor properties. The by-products of purine metabolic reprogramming are extensively engaged in tumor immune modulation, influencing the functions and recruitment of immune cells and molding an immune microenvironment that is propitious for tumor growth.

**Methods:**

We harnessed single-cell transcriptomics and spatial transcriptomics to concurrently analyze the purine metabolism (PM) features of the PC microenvironment. We quantitatively appraised the PM traits of diverse cell subsets via scoring algorithms such as AUCell and Ucell. Moreover, cell development and cell-cell interaction analysis elucidated the alterations in TME induced by PM dysregulation. Additionally, we defined the PM disorder characteristics of PC patients and utilized this to assess the immune phenotypes and prognoses of the patient population. Also, we identified the crucial intermediate genes that impact PM reprogramming and the establishment of an immunosuppressive environment within the TME of PC, and validated them through spatial sectioning and cell co-culture experiments.

**Results:**

Multi - dimensional transcriptome data elucidated the unique heterogeneity of PM in the PC microenvironment, which manifested that tumor cells and fibroblasts demonstrating higher PM scores in the TME. Cellchat analysis revealed that malignant cells with elevated PM expression were concomitantly associated with frequent interactions with CAFs as well as high expression of ligand-receptor pairs and transcription factors. Spatial data further corroborated this finding. Furthermore, the newly constructed PM disorder criteria indicated that patients with high PM levels were associated with a lack of response to immunotherapy and an immunosuppressive microenvironment. Finally, this study identified the singular role of NT5E in the immunosuppression resulting from PM reprogramming in PC. CCK8 and invasion experiments following the co-culture model demonstrated that intervention targeting NT5E could reverse the augmented malignancy of PC induced by co-cultured CAFs. NT5E is potentially a key target for reversing the “stiff-cancer” characteristics of PC.

**Conclusion:**

This study demonstrates that PM metabolic disorders could impinge upon tumor immunotherapy and exacerbate the immunosuppression engendered by the progression of PC fibrosis. Therapeutic strategies targeting PM or NT5E may offer a ray of hope for patients with advanced PDAC.

## 1 Introduction

Among all solid tumors, PC exhibits one of the gravest prognoses. It is characterized by highly intricate genomic features. The prevalence of KRAS mutations exceeds 90%, and mutations in genes like TP53, SMAD4, and CDKN2A are also rather common. Moreover, pancreatic ductal adenocarcinoma (PDAC) tissue is replete with a substantial amount of dense stroma, which consists of cancer-associated fibroblasts (CAFs), extracellular matrix (ECM), vascular endothelial cells, and immune cells (1–3). This structure assumes a pivotal role in tumor development. Functioning as a formidable “barrier,” it impedes the infiltration of immune cells into tumor cells, thereby hampering the immune system from exerting its normal anti-tumor function (4). Simultaneously, it exerts immunosuppressive effects, capable of suppressing the activity of immune cells and establishing an “immune-privileged zone” for tumor cells (5–7). This dense stroma is also intricately associated with treatment resistance, severely undermining the efficacy of diverse treatment modalities and enabling tumor cells to elude drug-mediated attacks (5). Immune Checkpoint Blockade (ICB) has demonstrated limited efficacy in the treatment of PDAC. PDAC is characterized by typical immune-cold tumor features, which represent one of the primary factors contributing to the meager efficacy of ICB (8–10). CAFs, being one of the principal cell components within the PDAC microenvironment, constitute 50%-80% of the tumor stroma (11, 12). Among them, iCAF plays a significant role in immunosuppression. It predominantly secretes immunosuppressive factors such as IL-6, IL-10, and CXCL12. These factors can recruit Tregs and M2-type macrophages into the tumor microenvironment while suppressing the functions of effector T cells (Teff), thus significantly attenuating the efficacy of ICB (12–14).

Tumor metabolic reprogramming constitutes a fundamental and pivotal mechanism through which cancer cells adapt to the dynamic changes within the tumor microenvironment (TME), thereby ensuring their survival and proliferation. This process is of utmost significance in the pathogenesis and progression of PDAC (15). PDAC cells display remarkable metabolic remodeling, and aberrations in glucose, glutamine, and lipid metabolism have been extensively investigated (16). Owing to their rapid proliferation, tumor cells have an escalated demand for purines. This renders the purine metabolic pathway hyperactive and transforms it into a potential crucial target for tumor therapy. In the realm of tumor treatment, intervention strategies targeting purine metabolism have exhibited certain promise. By inhibiting key enzymes involved in purine synthesis, such as phosphoribosyl pyrophosphate synthetase (PRPS), the purine supply to tumor cells can be disrupted, and their proliferation can be inhibited (17) Certain antimetabolic drugs, like methotrexate, exert their anti-tumor effects in accordance with this principle (17). Furthermore, purine metabolites are intricately involved in the regulation of the tumor immune response. Tumor cells can manipulate the functions and recruitment of immune cells by releasing purine metabolites, thereby creating an immune microenvironment that is permissive to tumor growth and progression (18). In recent years, there has been a growing interest in understanding the role of purine metabolism in the immune regulation of PDAC.

The purine metabolism pathway primarily encompasses the synthesis and degradation of adenine and guanine nucleotides. The products of this pathway serve multiple essential functions. Not only do they act as the fundamental building blocks for DNA and RNA synthesis, providing the necessary material basis for the rapid proliferation of tumor cells, but they also play a central role in energy metabolism (e.g., ATP and GTP) and signal transduction processes (e.g., cAMP and cGMP) (19). PDAC cells often upregulate the purine synthesis pathway to enhance their proliferative capacity. For instance, the overexpression of genes such as PRPS2, ATIC, PPAT, and IMPDH1 enables tumor cells to rapidly synthesize purines, thereby meeting the high demands of their continuous proliferation (20). Concurrently, this process also promotes immunosuppression. In the PDAC microenvironment, extracellular ATP secreted by tumor cells can be hydrolyzed into adenosine via the CD39/CD73 cascade. As a crucial immune-regulatory molecule, adenosine acts on effector T cells through A2AR, inhibiting their proliferation and cytotoxicity and preventing effector T cells from effectively fulfilling their anti-tumor function (20, 21). Moreover, adenosine can induce the differentiation of M2-type macrophages, which further promotes the formation of an immunosuppressive tumor microenvironment, allowing tumor cells to evade immune surveillance and continue to survive and progress (21–23).

Consequently, delving deeply into the molecular mechanism of purine metabolism in Pancreatic cancer (PC) is of utmost significance for elucidating the pathogenesis of PC, identifying novel therapeutic targets, and developing more efficacious combination treatment strategies. To this end, this study employed the most comprehensive spatial transcriptomics and single-cell transcriptomics technologies to analyze the purine metabolism characteristics of the PC microenvironment and sought to identify the key factors regulating these relationships. It is anticipated that through precise intervention in the purine metabolic pathway, the immunosuppressive microenvironment of PC can be disrupted, and the efficacy of immunotherapy can be enhanced.

## 2 Methods

### 2.1 Data acquisition and standardized processing

In the present study, the sequencing data of TCGA-PDAC along with the corresponding clinical data were all retrieved from the UCSC Xena website (https://xenabrowser.net/). The GEO database was harnessed to obtain single-cell sequencing data of PC patients, encompassing one normal pancreatic tissue sample sourced from GSE165399 and two PC patient tissue samples from GSE154778. To guarantee the accuracy and consistency of the data, the “limma” and “sva” R packages were employed to carry out batch calibration and integration on the sequencing gene expression datasets. The “harmony” algorithm was utilized to integrate single-cell sequencing samples, thereby eliminating potential batch effects. The GeneCards database was utilized to amass the “purine metabolism” gene set, which was subsequently applied for scoring and gene screening in the ensuing research.

### 2.2 Identification of cell sub-populations in PC single-cell data and determination of purine metabolism criteria

The “Seurat” R package was utilized to conduct pre-processing on the scRNA-seq data. The “PercentageFeatureSet” function was employed to assess the proportion of low-quality genes within the dataset. Each cell was requisite to express a gene count ranging from 200 to 10,000, and the mitochondrial content was required to be less than 20%. Subsequent to the aforementioned screening, the “NormalizeData” function was used to standardize the scRNA- seq data. The standardized data was then converted into a Seurat object, and the “FindVariableFeatures” function was utilized to identify highly variable genes. The “RunPCA” tool was employed to scale and execute principal component analysis on this set of highly variable genes. To more effectively present the data characteristics, the data underwent dimensionality reduction and visualization through algorithms. The “FindAllMarkers” and “FindMarkers” algorithms were utilized to perform Wilcoxon tests, meticulously compare different cell types, and annotate diverse cell sub-populations in accordance with these marker genes. To evaluate the purine metabolism characteristics of the single-cell sequencing data of PC, five commonly-used algorithms were adopted, namely “AddModuleScore”, “ssGSEA”, “AUCell”, “UCell”, and “singscore”. Bubble plots were utilized to visualize the purine metabolism characteristics of each individual cell.

### 2.3 Inference of cell developmental trajectories and analysis of cell-cell interactions

The “Monocle” R package was utilized to meticulously analyze the pseudo-time trajectories of tumor cells. The “PLOT_CELL_TRACTURE” function was employed to precisely order and visualize different cell sub-populations in accordance with the pseudo- time sequence. The “CellChat” package was used to construct a ligand-receptor-level regulatory network grounded in the PC microenvironment. The “netVisualDiffInteraction” algorithm was utilized to compare the intensity of cell-cell communication.

### 2.4 Processing of PC spatial sections and annotation of spatial cell sub – populations

PDAC_GSE203612 and PDAC_GSE211895 were loaded from the GEO database as validations for the spatial transcriptome sections. The “SCTransform” within the Seurat package was utilized to standardize the UMI counts. “RunPCA” was employed for dimensionality reduction and unsupervised clustering analysis. With the assistance of the “SpatialFeaturePlot” function, the visualization of subgroups and genes was accomplished. “stLearn” developed based on “Scanpy” was utilized for spatial transcriptome data analysis to determine the gene expression characteristics of cells in disparate regions and unearth the interactions and spatial distribution patterns of cells within the tumor microenvironment. “RCTD” was utilized in this study to discriminate the gene expression characteristics of different cell types such as tumor cells, immune cells, and stromal cells.

### 2.5 Establishment of PM criteria and evaluation of clinical characteristics

The TCGA cohort was randomly partitioned into a training set and a validation set. The R software package “survival” was utilized to perform univariate Cox regression analysis to screen out genes that were statistically significant with respect to the prognosis of PDAC patients. Subsequently, Lasso and multivariate step-wise regression analyses were carried out to ascertain the most critical combination of factors influencing the prognosis. A PM score was computed and assigned to each PDAC patient, and patients were classified into high- risk and low-risk groups based on the median PM score. The Kaplan-Meier method was employed to understand the survival status of patients, and the ROC curve was introduced to gauge the prediction performance of the model. A column-line graph was constructed to calculate the 1, 3, 5 years overall survival rates of PDAC patients, with risk scores, age, and clinical stages incorporated as independent prognostic factors.

### 2.6 Correlation analysis between PM score and immune characteristics

The R package “Estimate” was utilized to estimate the abundances of tumor mesenchymal and immune cells in PC patients and concurrently evaluate tumor purity. The results of seven methods for evaluating immune infiltration were procured from the TIMER 2.0 database. ssGSEA was utilized to score specific characteristic gene sets to understand the activity levels of different immune cell types within the samples. Additionally, the tumor stem cell index obtained from prior studies was used to quantify the stem cell characteristics of tumor samples. To evaluate the likelihood of immune escape in tumor samples, we adopted the TIDE calculation framework (http://tide.dfci.harvard.edu/) to assess the immune escape status of tumor samples. Moreover, “ggplot2” was utilized to present the data of PM score and TMB in the form of scatter plots and line graphs.

### 2.7 Cell culture and establishment of co-culture system

In this study, the PC cells (PANC-1) utilized in the experiment were procured from the American Type Culture Collection (ATCC), and the human pancreatic tumor fibroblasts (immortalized) were obtained from the Chinese Academy of Sciences. Both types of cells were cultured in PRMI1640 medium supplemented with 10% high-quality fetal bovine serum. Subsequent to operations such as passage and stable transfection, the aforementioned cells were co-cultured in a transwell mold to observe the survival-related effects of inter-cellular signaling factors on the two types of cells.

### 2.8 sh-NT5E cell transfection

The sh-LY6D knockdown plasmid from GenePharma Company was selected. The cells to be treated were inoculated in a 6-well plate. When the cell confluence reached 50%, Lipofectamine 3000RNAiMAX was utilized for the transfection procedure.

### 2.9 CCK-8 cell viability measurement experiment

The cells were prepared into a homogeneous suspension and precisely inoculated into a 96- well plate at a density of 5×10³ cells per well and incubated for 24 hours. Subsequently, 10 μL of CCK-8 labeling agent was added to each well. After the addition of the labeling agent, to avert the influence of light on the reagent, the 96-well plate was placed in a 37°C environment and incubated in the dark for an additional 2 hours. Thereafter, a microplate reader was used to continuously measure the absorbance values at 450nm, and the alteration in absorbance was employed to mirror the cell viability.

### 2.10 Cell invasion experiment

The PANC-1 cells were pre-treated with starvation to render the cells in a relatively quiescent state. Post-treatment, the cell suspension was added to the upper chamber containing Costar, and a serum-containing medium was added to the lower chamber. Subsequently, it was placed in an incubator for 48 hours to afford the cells sufficient time for invasion activities. After the experiment, the cells were fixed with 4% paraformaldehyde, stained with crystal violet, and then subjected to counting and analysis.

### 2.11 Statistical analysis

All data calculation steps involved in this article were carried out using the 64-bit R version 4.2 and its accompanying publicly available R packages. The specific statistical verification rules were defined during the development process of the R packages. In this study, an asterisk (*) represents P < 0.05, which is considered to be statistically significant.

## 3 Results

### 3.1 Comparison of purine metabolism characteristics in the PC microenvironment via multiple algorithms

**Figure 1:**
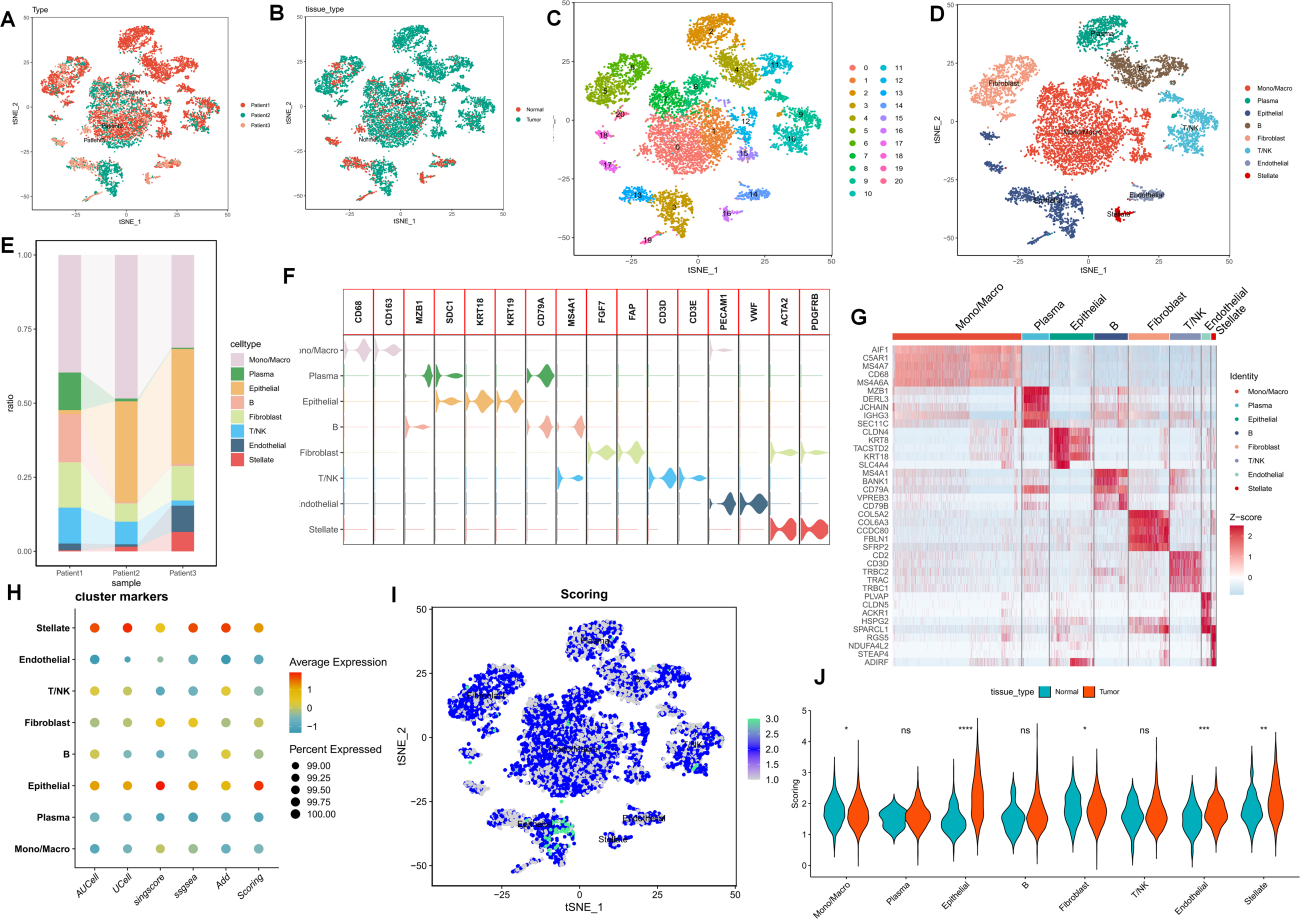
**(A-E)** The UMAP plot elucidates the clustering pattern of the samples. **(F, G)** The expression profiles of specifically-expressed marker genes within diverse cell clusters are presented. **(H)** Five single-cell scoring tools are employed to quantitatively assess the PM characteristics of distinct cell clusters. **(I, J)** A comparison of PMscores between tumor samples and normal samples is conducted. p values < 0.05 (*p < 0.05) were considered significant, where **p < 0.01, ***p < 0.001, and ****p < 0.0001, ns (non- significant).

Following quality control and dimensionality reduction of paired samples from three PC patients, the present study clustered into the subsequent key cell clusters: mononuclear macrophages, plasma cells, epithelial cells, T/NK cells, B cells, endothelial cells, fibroblasts, plasma cells, and pancreatic stellate cells (**Figures 1A–E**). The cellular composition within the PC microenvironment across different patients exhibited relative equilibrium, and the specifically-expressed marker genes of diverse cell clusters are presented in (**Figures 1F, G**). To comprehensively analyze the purine metabolism characteristics in the PC microenvironment, the current study utilized five single-cell scoring tools, namely AddModuleScore, UCell, GSVA, AUCell, and singscore, for assessment. The scores assigned to different cells demonstrated variability. Overall, endothelial cells and stellate cells within the PC cell microenvironment manifested higher purine metabolism scores, which we hereinafter referred to as PMscore (**Figure 1H** **).** Subsequently, a comparison of PMscores between tumor and normal samples was conducted. The findings revealed disparities in endothelial cells, epithelial cells, stellate cells, and macrophages, with epithelial and stellate cells showing distinct differences between normal and cancerous samples (**Figures 1I, J**).

### 3.2 Imbalance in cell differentiation and interaction induced by abnormal purine metabolism

**Figure 2:**
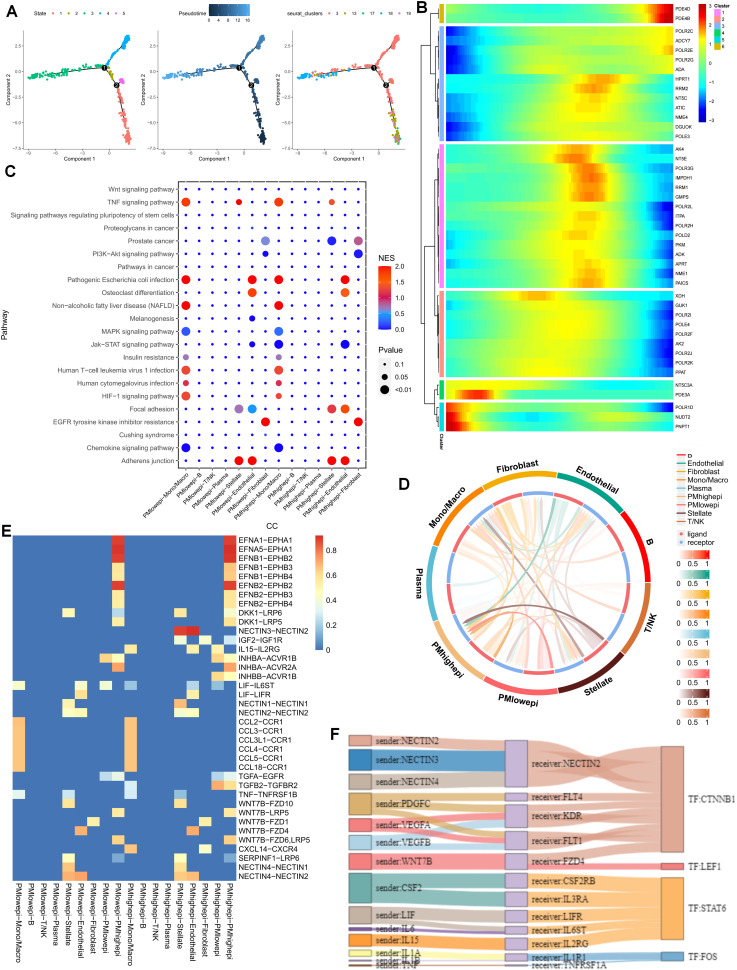
**(A)** Cell trajectory analysis was executed on epithelial cell clusters. **(B)** A heatmap illustrates that different purine-metabolism genes are also in a dynamic state during the process of cell differentiation. **(C)** A comparison of common signaling pathways between the PMS low group and the PMS high group is carried out. **(D)** A comparison of cell-cell interactions between the PMS low group and the PMS high group is performed. **(E, F)** A comparison of ligand-receptor relationships between the PMS low group and the PMS high group is presented.

To gain a more lucid understanding of the impact of purine metabolism on the development of microenvironment cells, this study initially performed cell trajectory analysis on distinct epithelial cell clusters subsequent to dimensionality reduction. The epithelial cell states were classified into five distinct states, with cluster 18 representing the endpoint of cell differentiation (**Figure 2A**). Subsequently, the expression of purine-metabolism-related genes displaying the highest differential expression at various developmental stages of these cell clusters was depicted in a heat map. The results indicated that different purine-metabolism genes also underwent changes during the process of cell differentiation (**Figure 2B**). Further, epithelial cells were segregated into a PMS low group and a PMS high group based on the median PMS of epithelial cells in tumor tissues, and common signaling pathways were contrasted (**Figure 2C**). It was discerned that common signaling pathways such as the TNF signaling pathway and the HlF-1 signaling pathway exhibited significant differences between fibroblasts and stellate cells in different PMS groups, suggesting that this might represent a pivotal factor influencing the crosstalk among these three cell groups. Consequently, our focus shifted to cell-cell interactions. The results indicated that epithelial cells with high PMS received an increased number of signals from fibroblasts and stellate cells. Correspondingly, these cells transmitted more signals to fibroblasts, stellate cells, and T cells (**Figure 2D**). The ligand-receptor relationships within this process were presented, with the NECTIN3-NECTIN2 signal and the WNT7B-FZD1 emerging as the most prominent ligand-receptor relationships in the interaction between these two cell groups (**Figures 2E, F**). To further enrich the potential cell-mechanism network, we identified two transcription factors, CTNNB1 and LEF1, which may be implicated in regulating the interaction between these two cell groups and could potentially serve as a crucial means of restoring the purine metabolism imbalance in the PC microenvironment in the future (**Figures 2E, F**).

### 3.3 Unveiling cell disorders resulting from purine metabolism imbalance from a spatial perspective

**Figure 3:**
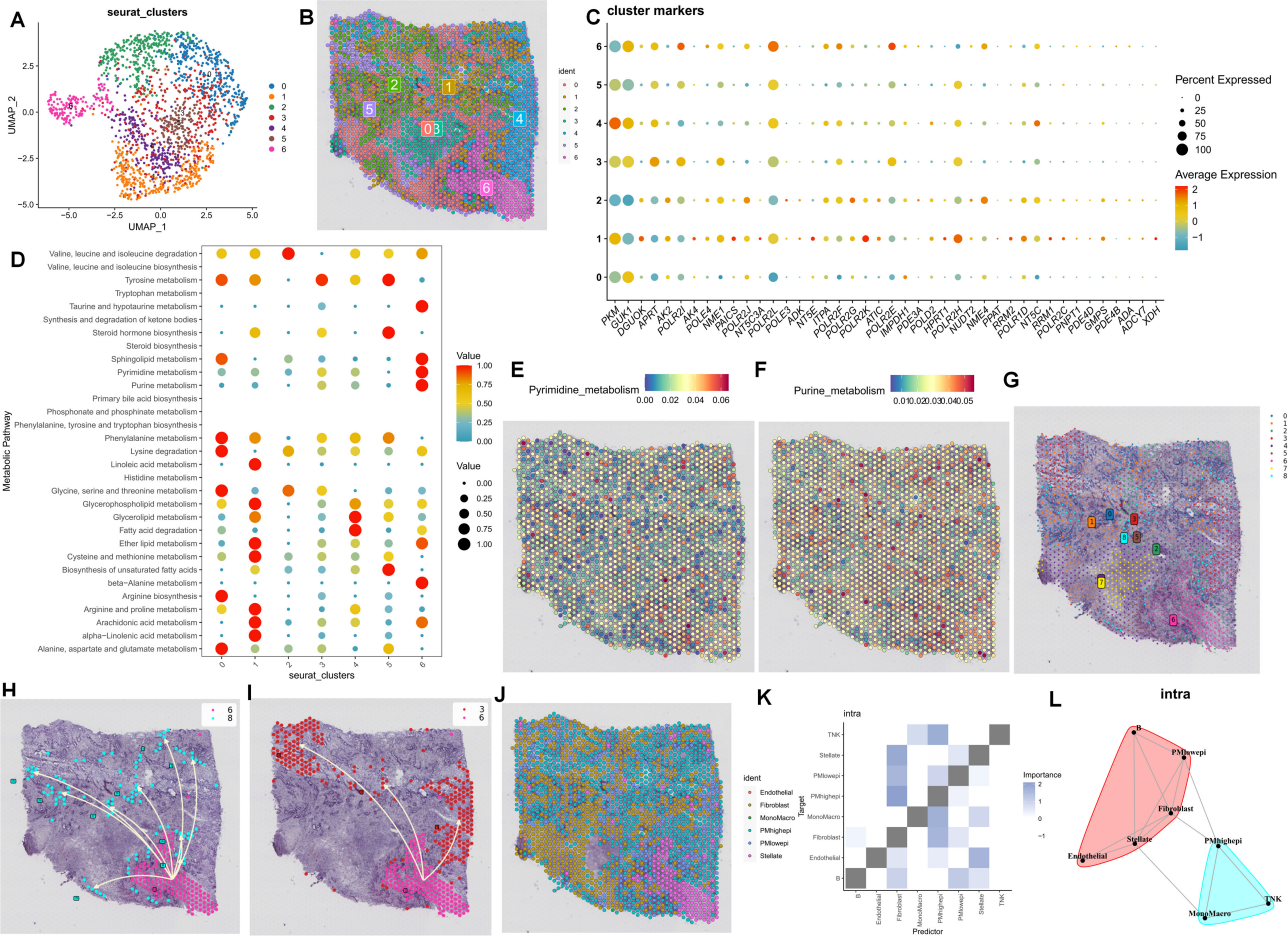
**(A, B)** Six distinct cell sub-populations were identified through the joint annotation of spatial transcriptome sections and single-cell data. **(C)** The expression patterns of the most significant genes in purine metabolism at various spatial spots are shown. **(D)** The metabolic activities in different regions are analyzed. **(E, F)** Heatmaps depicting sphingolipid metabolism and taurine metabolism in spatial sections are presented. **(G-I)** The developmental trajectories of cell sub-populations from a spatial perspective are investigated. **(J-L)** Spatial-location interaction is utilized to quantitatively analyze the relationships between different cell clusters.

As previously mentioned, the PDAC tumor microenvironment experiences alterations in cell communication and differentiation in the presence of purine-metabolism imbalance. To more precisely delineate this characteristic, we utilized spatial transcriptome sections to outline this aspect. Initially, six distinct cell sub-populations were identified in the PC spatial sections, primarily facilitated by the joint annotation of single-cell data (**Figures 3A, B**). Genes of utmost significance in purine metabolism exhibited varying expressions at different spatial locations. Genes such as PKM and GUK1 demonstrated substantial differences across different clusters, indicating their potential involvement in the tandem interaction between spatial spots (**Figure 3C**). To comprehensively assess the metabolic disorders within the tumor microenvironment, we further employed the “scMetabolism” R package to analyze the metabolic activities in different regions. The results revealed a close association between Cluster 6 and purine-metabolism activity (**Figure 3D**). Additionally, we observed that this region was concomitantly accompanied by active sphingolipid metabolism and taurine metabolism (**Figures 3E, F**). From a spatial perspective, upon further examination of the developmental trajectories of cell sub-populations, cells in region 6 with pronounced purine metabolism were found to be undergoing differentiation towards region 3 and region 8 (**Figures 3G–I**). We further utilized the RCTD algorithm to map cells onto the spatial framework. The results indicated that epithelial cells with high PMS were diffusely distributed within the tumor microenvironment of PC, a characteristic associated with the unique “stiff-cancer” phenotype of PC, suggesting that high purine metabolism may offer a direction for overcoming this phenotype (**Figure 3J**). Subsequently, we quantitatively analyzed the relationships between different cell clusters using spatial-location interaction. The results corroborated previous findings, with epithelial cells having high PMS showing a significant correlation with fibroblasts (**Figures 3K, L**).

### 3.4 Construction of the purine-metabolism disorder index PMS for observing the clinical characteristics of the PDAC population

**Figure 4:**
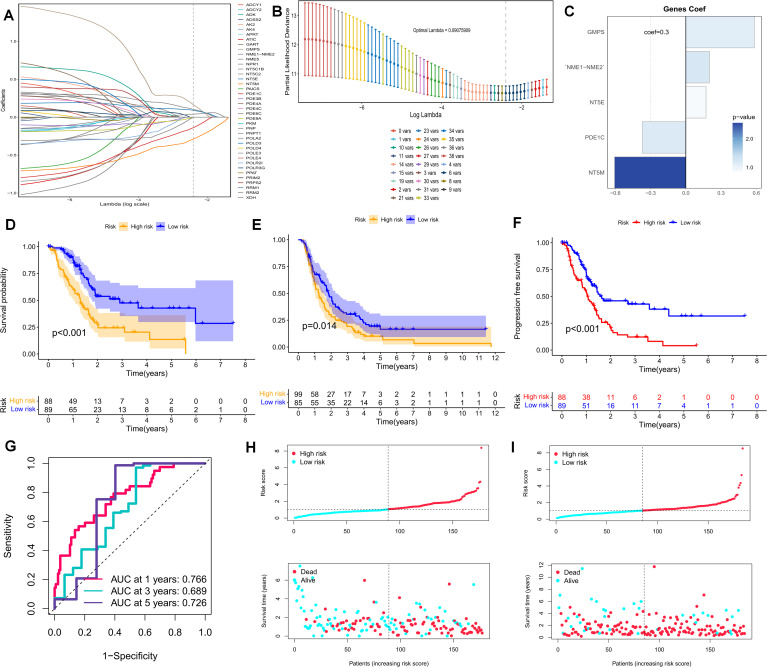
**(A, B)** Lasso regression analysis was performed on the 40 genes obtained subsequent to univariate COX regression analysis. **(C)** The Coef values of the model-building genes are determined. **(D, F)** The discrimination of PMS and the prognoses of patients in the validation set and the training set are evaluated. **(F)** The PFS of patients in the training set is analyzed. **(G)** The standard classification based on purine-metabolism disorder can effectively predict the AUC curve of pancreatic cancer patients. **(H, I)** The risk plots of each patient in the training set and the validation cohort are presented.

**Figure 5:**
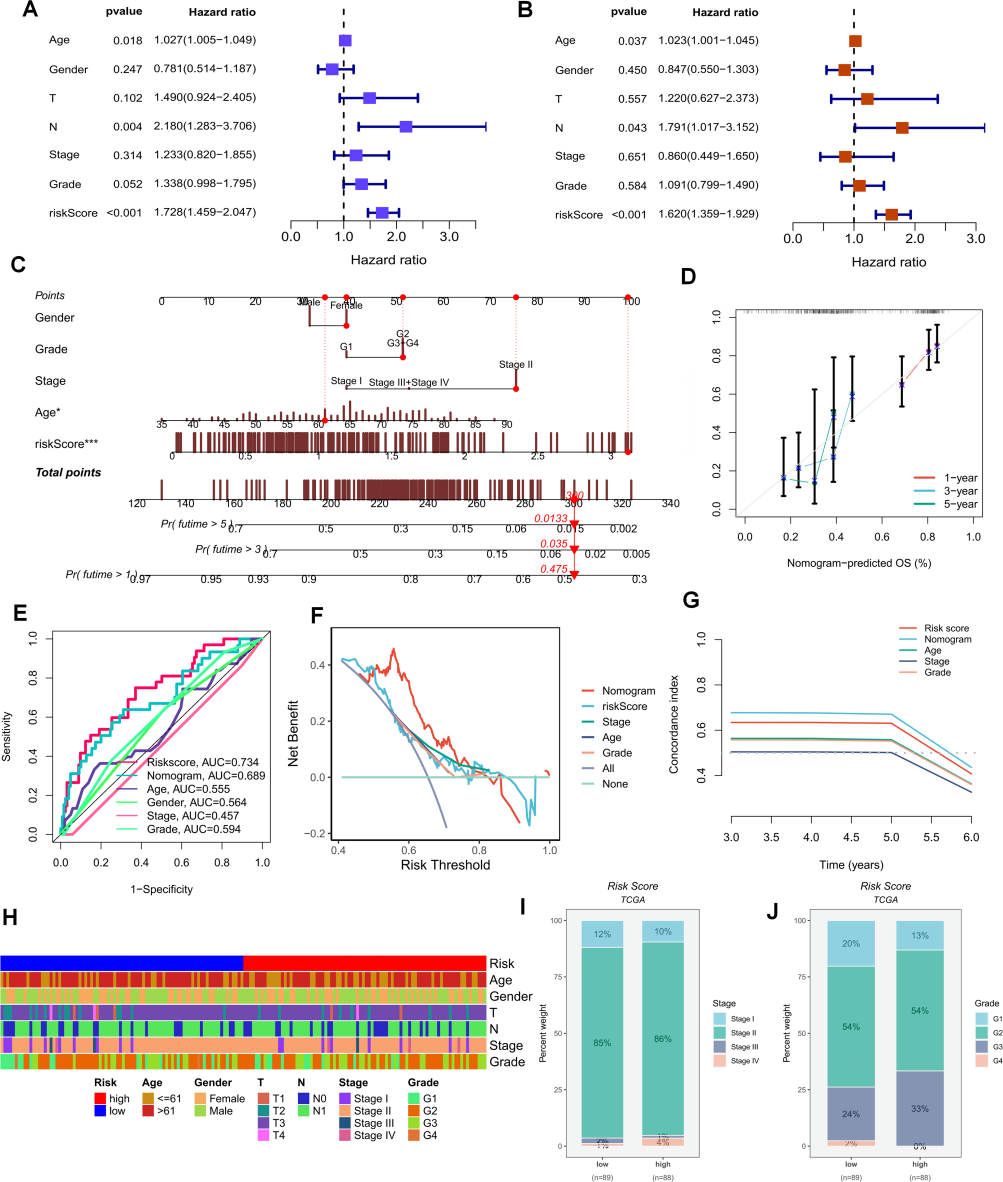
**(A, B)** Univariate and multivariate Cox analyses demonstrate that PMS can be utilized as an independent prognostic indicator for PAAD patients. **(C, D)** Nomograms for predicting the survival rates of PAAD patients at different time intervals are constructed. **(E–G)** The DCA curve and C-index value reveal the current nomogram constructed based on PMS. **(H)** The differences in clinical parameters between the groups of people with high and low risk scores. **(I, J)** The correlations between the risk scores and the clinical and pathological stages of the tumors.

The foregoing clues suggest that purine metabolism may instigate disorders within the PDAC tumor microenvironment. Consequently, we constructed a purine-metabolism disorder index to investigate the purine-metabolism disorder characteristics of the PC patient population. The purine-metabolism gene set was intersected with the differential genes of epithelial cells exhibiting high and low PMS expressions. Subsequently, lasso regression analysis was performed on the 40 genes obtained following univariate COX regression analysis (**Figures 4A, B**). Taking into account over-fitting and other relevant factors, and narrowing the gene range for predicting OS, a 5-gene signature was constructed through Lasso-Cox regression analysis. Based on PMS, with the median serving as the cut-off point, patients were categorized into a low-PMS group and a high-PMS group. The risk plot demonstrated that, within both the training and validation groups, the OS of patients in the high-PMS group was inferior to that of patients in the low-PMS group (**Figures 4C–F**). Moreover, the AUC curve in this study indicated that the classification criterion based on purine-metabolism disorder could effectively predict the prognosis of PC patients (**Figure 4G**). This performance was further accentuated in the risk plot, which presented the detailed survival outcomes of each patient in the training set and the validation cohort (**Figures 4H, I**). Through a comprehensive comparison of univariate and multivariate Cox analyses, PMS was identified as an independent prognostic indicator for PDAC patients, with the area under the curve (AUC) of PMS being considerably higher than that of other clinicopathological features (**Figures 5A, B**). A nomogram developed for predicting the survival rates of PDAC patients at different time intervals demonstrated that PMS could yield accurate predictions (**Figures 5C, D**). Additionally, the DCA curve and C-index value indicated that the current nomogram constructed based on PMS provided the highest net benefit, representing an optimization of traditional models in the context of clinical decision-making (**Figures 5E-G**). Simultaneously, the current PMS was found to be strongly associated with clinical-stage characteristics. Specifically, patients with high PMS were frequently in the advanced stage of PDAC (**Figures 5E–G**).

### 3.5 Influence of purine-metabolism disorder on immune infiltration in PDAC patients

**Figure 6:**
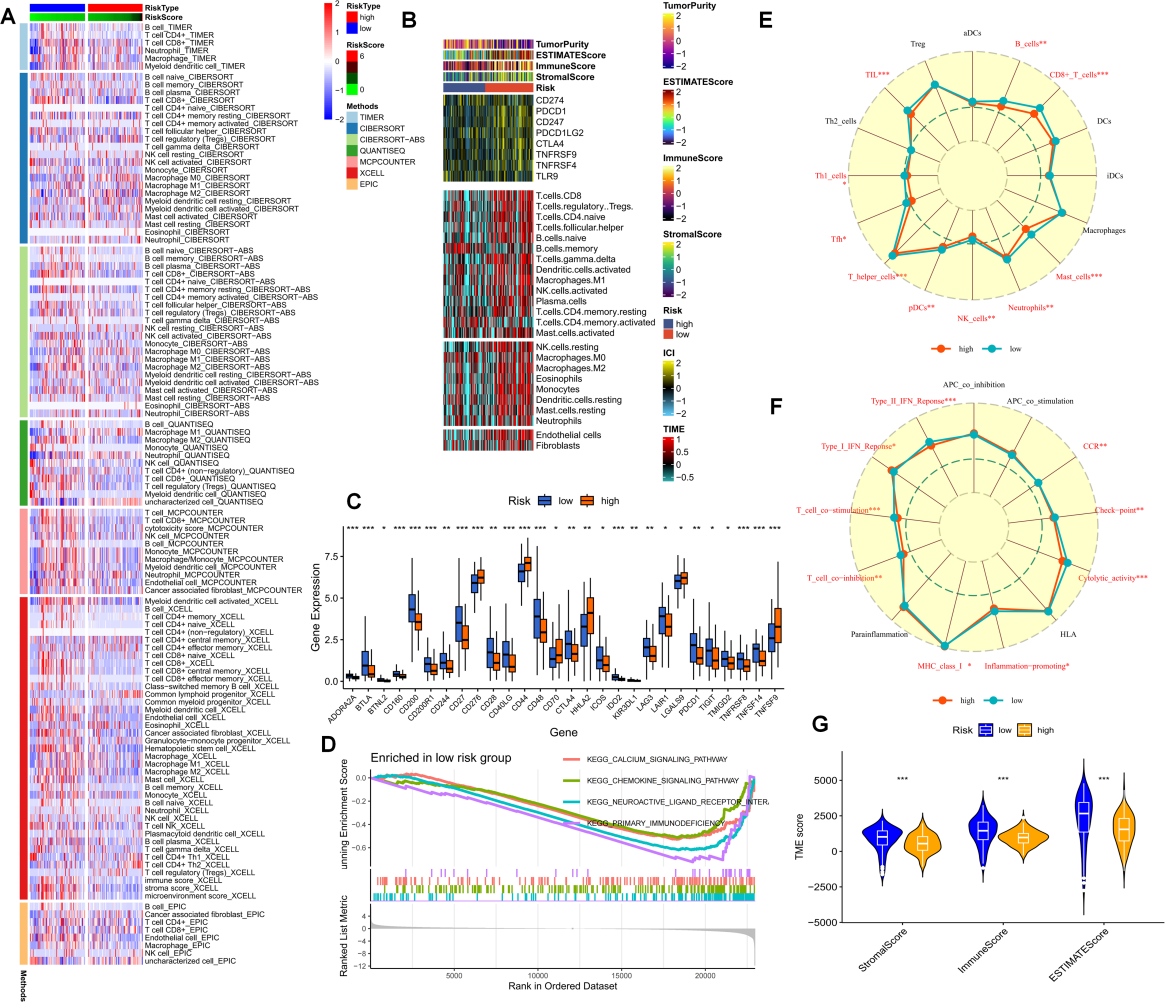
**(A)** The immune-cell infiltration in different PMS populations is examined. **(B)** A heatmap portrays the immune-checkpoint genes, immune scores, immune-cell infiltration, and tumor-microenvironment scores in different PMS groups. **(C)** The expression levels of immune-checkpoint molecules in the PMS low group and the PMS high group are compared. **(D)** GSEA results further indicate that high PMS is accompanied by the inhibition of immune pathways. **(E-G)** The ssGSEA method is employed to evaluate the enrichment scores of different immune-cell subsets and functions. p values < 0.05 (*p < 0.05) were considered significant, where **p < 0.01, ***p < 0.001, and ****p < 0.0001).

As previously stated, purine-metabolism disorder may disrupt the cell-composition components of the PDAC tumor microenvironment. The TME has been established as a critical determinant of patients’ clinical outcomes and immunotherapy responses. Hence, this study delved deeper into the immune-cell infiltration in different PMS populations. The reliability of this aspect was enhanced through cross-validation using multiple immune-infiltration algorithms. Specifically, the low-PMS population typically exhibited heightened activation of immune-effector cells, including T cells, NK cells, and B cells (**Figure 6A**). Subsequently, we depicted the immune-checkpoint genes, immune scores, immune-cell infiltration, and tumor-microenvironment scores in different PMS groups via a heat map (**Figure 6B**). The results indicated that patients in the low-PMS group had reduced immune activation, suggesting the presence of an immunosuppressive microenvironment in this subset of patients (**Figure 6B**). Consequently, we considered the potential impact of immune-checkpoint molecules on patients with purine-metabolism disorders, observing that the expression levels of the vast majority of immune checkpoints were elevated in the high-PMS group (**Figure 6C**). Additionally, GSEA results indicated that high PMS was associated with the inhibition of immune pathways (**Figure 6D**). More specifically, we employed the ssGSEA method to evaluate the enrichment scores of different immune-cell subsets and functions, aiming to gain a more in-depth understanding of the relationship between the risk score and immune-related functions. The results suggested that the immune-cell infiltration scores and immune-pathway scores in the high-PMS group were significantly lower compared to those in the low-PMS group (**Figures 6E–G**). Overall, the above results indicated that the low-PMS group often displayed more immune-activation characteristics, as further corroborated by the immune scores, suggesting that modulating purine-metabolism disorders may provide crucial evidence for enhancing immunotherapy (**Figures 6E–G**).

### 3.6 Association of high PMS population with low response to immunotherapy

**Figure 7:**
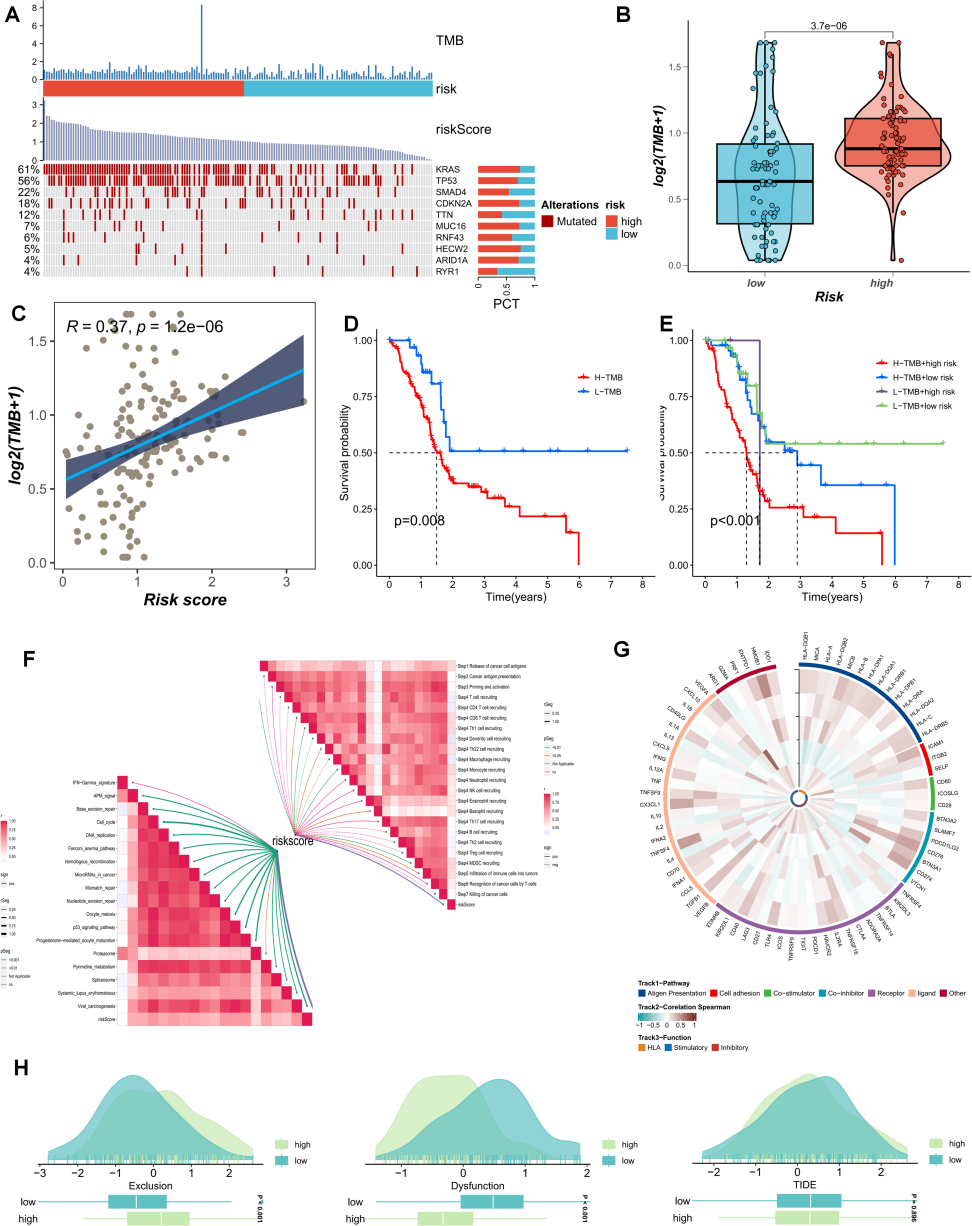
**(A)** The distribution of common mutated genes in PAAD patients across risk-score subgroups is analyzed. **(B)** The TMB mutation levels in the PMS low group and the PMS high group are compared. **(C)** The correlation between PMS score and TMB is explored. **(D, E)** The survival outcomes of patients divided into four groups according to the median TMB value and PMS value are investigated. **(F)** The association between PMS and the tumor-immune cycle is analyzed. **(G-H)** Correlation analysis of model genes and classic immune-related genes is conducted to study the differences in immune responses between different subgroups.

The current study posits that a higher TMB generally implies that tumor cells harbor a greater number of gene mutations, rendering them more readily recognizable by the immune system. We analyzed the distribution of the most prevalent mutated genes in PDAC patients across risk-score subgroups (**Figure 7A**). The high-PMS population was frequently associated with a greater number of mutations in KRAS, TP53, and SMAD4 (**Figure 7A**). Additionally, this subgroup exhibited more TMB mutations, with the TMB mutation level being significantly higher than that of the low-PMS population (**Figure 7B**). Accordingly, patients were divided into four groups based on the median TMB and PMS values. The results indicated that the OS of patients with high PMS and high TMB was relatively poor, which we attribute to the more pronounced immunosuppression in high-PMS patients (**Figures 7C–E**). Moreover, PMS exhibited substantial and meaningful associations with numerous key stages of the tumor-immune cycle (**Figure 7F**). Subsequently, we conducted a correlation analysis involving model genes and classic immune-related genes to explore the differences in immune responses among different subgroups (**Figures 7G–H**). The TIDE prediction score is currently regarded as a reliable metric for evaluating the reactivity of immune-checkpoint blockade (ICB). Consequently, we conducted a PMS-correlation analysis of the TIDE score and the immune-exhaustion score. The current research findings revealed that individuals in the high-PMS group exhibited higher Immune Exclusion and lower Immune Dysfunction scores, although the difference in TIDE scores between the two groups was not particularly significant (**Figures 7G–H**).

### 3.7 Identification of key representative genes influenced by purine-metabolism disorder in immunotherapy

**Figure 8:**
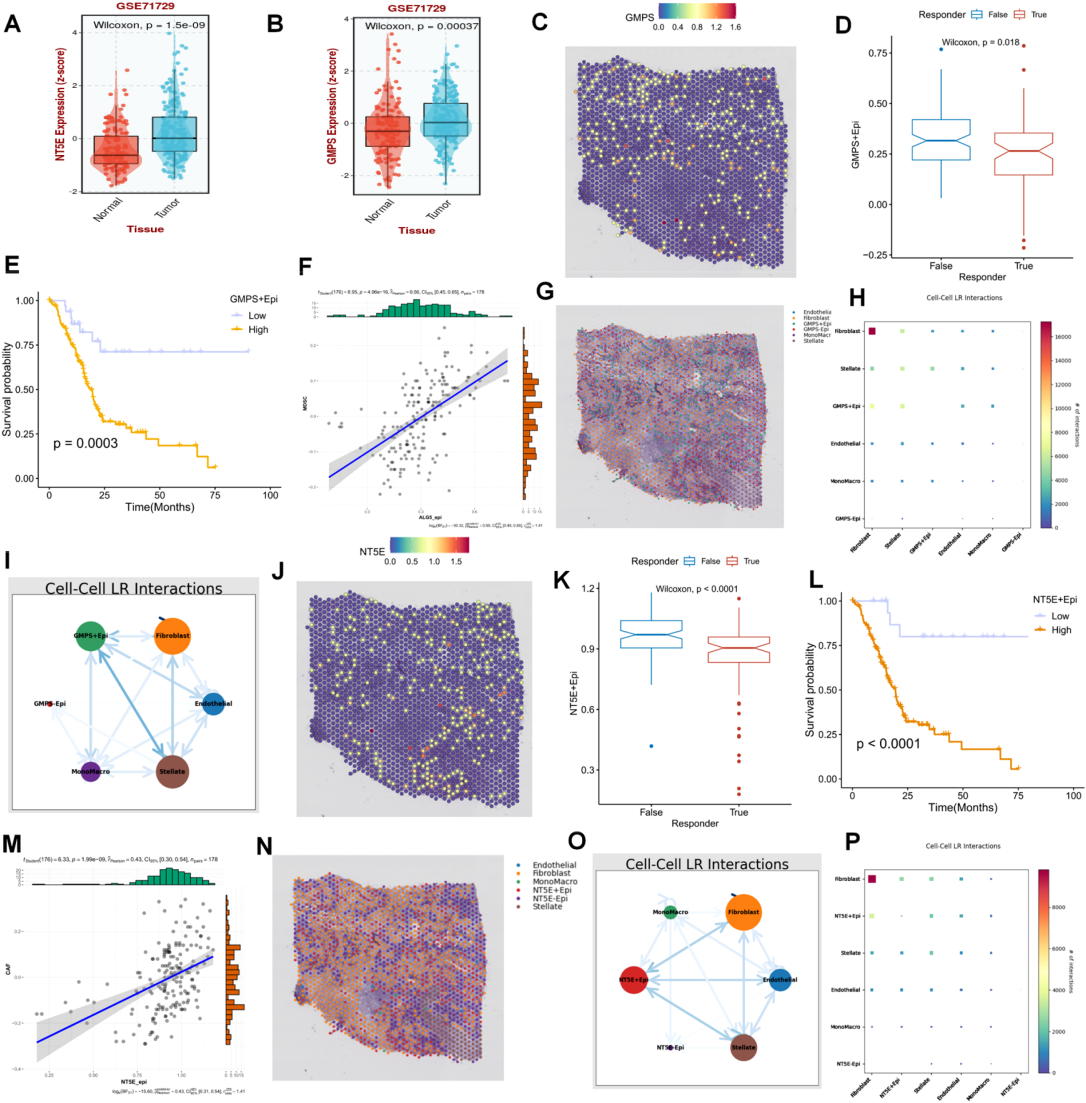
**(A, B)** Two key representative genes, GMPS and NT5E, were identified. **(C)** The expression of GMPS on spatial sections is visualized. **(D)** The population with high GMPS expression exhibits a poorer response to immunotherapy. **(E)** The prognosis of the population with high-expressing GMPS cells is significantly adverse. **(F-I)** Cells with high GMPS expression are significantly correlated with MDSC cells. **(J)** The expression of NT5E on spatial sections is presented. **(K)** The population with high NT5E expression shows a poorer response to immunotherapy. **(L)** The prognosis of the population with high-expressing NT5E cells is significantly unfavorable. **(M-P)** Cells with high NT5E expression are significantly associated with MDSC cells.

Following preliminary screening, the present study identified two key representative genes, GMPS and NT5E, both of which were highly expressed in the PC population relative to the normal population (**Figures 8A, B**). On spatial sections, the expression of GMPS was diffusely distributed within the tumor area, and the population with high GMPS expression typically demonstrated a poorer response to immunotherapy (**Figures 8C, D**). Additionally, the prognosis of the population with high expression of these cells was also significantly unfavorable (**Figure 8E**). To elucidate this phenomenon from the perspective of crosstalk within the PDAC immune microenvironment, it was predominantly observed that cells with high GMPS expression were significantly associated with MDSC cells (**Figures 8F–I**). When focusing on NT5E, we found that NT5E+ cells were more malignant in terms of both prognosis and response to immunotherapy (**Figures 8J–P**). From a spatial analysis, it was observable that NT5E+ cells frequently engaged in more interactions with fibroblasts. In terms of spatial distribution, NT5E+ cells were diffusely distributed throughout the PC microenvironment alongside tumor-associated fibroblasts (**Figure 8J**). The cell-cell interactions based on spatial sections also indicated a significant interaction between the two (**Figures 8K–P**). Based on these results, our study posits that NT5E+ cells may play a pivotal role in the dysregulated microenvironment of PDAC fibroblasts following purine-metabolism disorder, with this role being more evident in the formation of an immunosuppressive microenvironment and a reduced response to immunotherapy (**Figures 8K–P**).

### 3.8 NT5E as an intermediate mediator in the purine-metabolic reprogramming of the PC TME and the formation of an immunosuppressive environment

**Figure 9:**
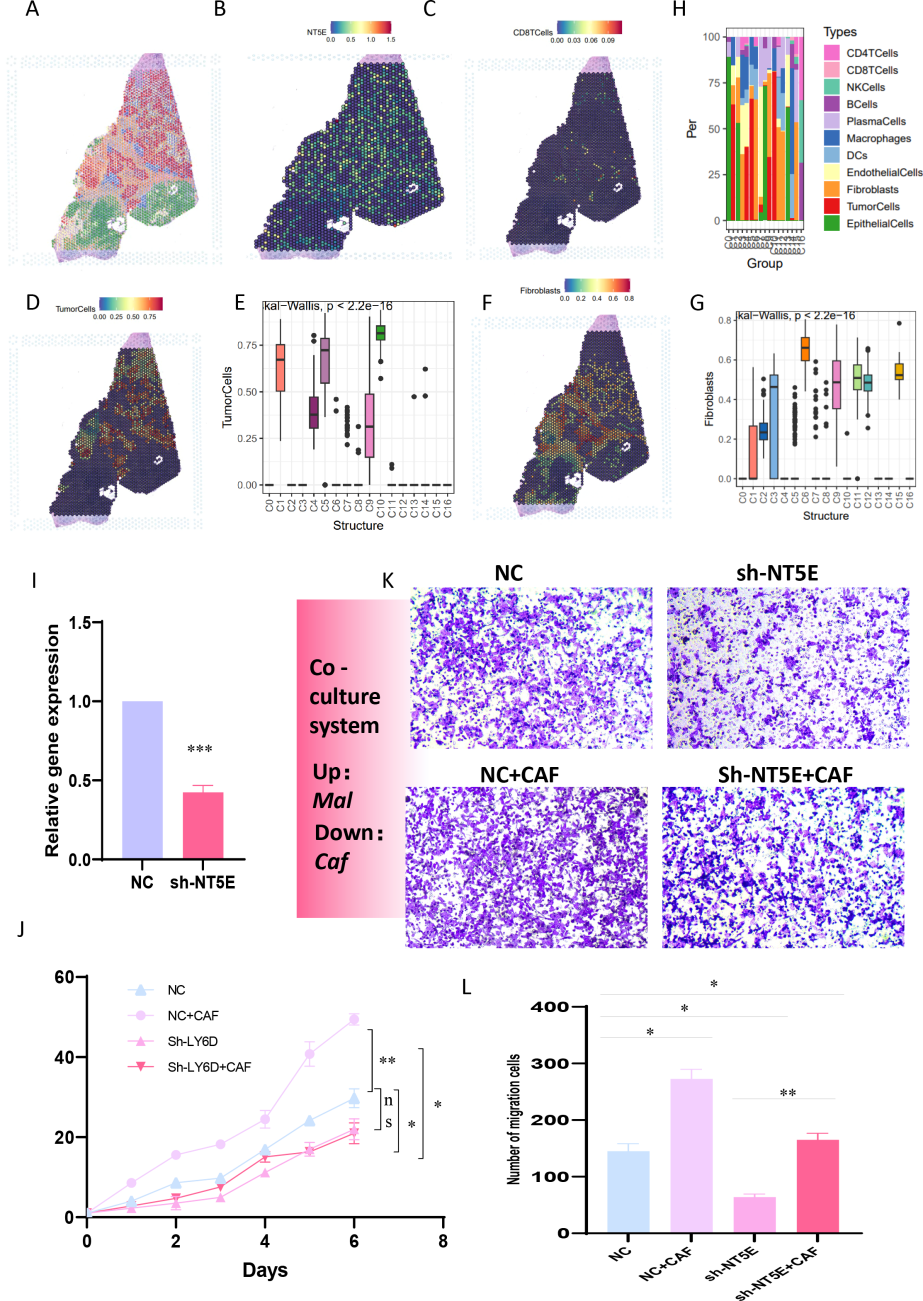
**(A-G)** The relationships among tumor cells, fibroblasts, T cells, and NT5E expression in spatial transcriptome sections are explored. **(H)** A bar chart depicts the cell distribution relationships within different spatial clusters. **(I)** Pancreatic cancer cells PANC-1 with stably transfected and knocked-down NT5E are prepared. **(K, L)** Transwell experiments are conducted to compare the invasion ability of pancreatic cancer cells in the co-culture system. **(L)** CCK-8 experiments are carried out to compare the survival ability of pancreatic cancer cells in the co-culture system. p values < 0.05 (*p < 0.05) were considered significant, where **p < 0.01, ***p < 0.001, and ****p < 0.0001, ns (non- significant).

To further reinforce the conclusions drawn above, we verified the relationships among tumor cells, fibroblasts, T cells, and NT5E expression in an additional spatial-transcriptome section. Following dimensionality reduction and clustering of the spatial sections, a total of 17 spatial cell clusters were obtained. Tumor cells were predominantly located in C1, C4, C5, C9, and C10, while fibroblasts were mainly distributed in C2, C3, C6, C9, C11, C12, and C15 (**Figures 9A–G**). By comparing the spatial positions, it was evident that NT5E was predominantly expressed on tumor cells, with fibroblasts interspersed among tumor cells, creating a typical “stiff-cancer” environment in PC, characterized by a scarcity of CD8 T cells (**Figures 9C, H**). By further analyzing the relationship between NT5E and tumor immunity from the bulk RNA-seq data, we can conclude that NT5E has the closest relationship with the infiltration of neutrophils, macrophages, and fibroblasts. Additionally, NT5E is also closely related to immune factors such as CCL13 and CCL18. This indicates that NT5E may play a mediating role in the tumor immune microenvironment of PC (**Supplementary Figures 1A, B**). These findings support the hypotheses presented in previous sections, namely, that the disorder and reprogramming of purine metabolism are predominantly manifested in tumor cells and fibroblasts, and that NT5E, a PM-disorder gene, indicates a poor prognosis for tumor patients. Consequently, we postulate that NT5E serves as an intermediate mediator in the purine-metabolic reprogramming of the PC TME and the formation of an immunosuppressive environment. To this end, we verified the co-culture model system. After stably transfecting and knocking down NT5E in PC cells PANC-1, they were co-cultured with pancreatic-cancer-associated fibroblasts (**Figure 9I**). The results suggested that knocking down NT5E could attenuate the growth and invasion capabilities of tumor cells; however, when co-cultured with CAFs, this trend was mitigated, indicating that the inter-cellular signal communication between CAFs and tumor cells plays a significant role in the malignant progression of PC (**Figures 9K–L**). In fact, our study further analyzed the significance of NT5E in the four immunotherapy datasets of GSE67501, GSE136961, GSE140901, and GSE165252. The results showed that NT5E could effectively distinguish the effects of immunotherapy. This indicates that the level of NT5E expression has a significant impact on the response of pancreatic cancer patients to immunotherapy. From another perspective, it also confirms the prominent role of NT5E in the immune microenvironment (**Supplementary Figures 1C–F**).

## 4 Discussion

Within the highly distinctive immunosuppressive microenvironment of PC, fibroblasts assume a role of utmost significance and centrality. PC, widely renowned as a “cold tumor”, which possess less infiltration of effector immune cells such as T cells, making it difficult for them to effectively recognize and attack tumor cells, owes this designation to its uniquely fibrotic microenvironment. The establishment of its immunosuppressive characteristics is intricately intertwined with the functional remodeling of cancer-associated fibroblasts (CAFs) (10). Notably, CAFs not only erect a physical barrier through the secretion of extracellular matrix (ECM) proteins, thereby impeding the infiltration of T-cells, but also release immunosuppressive factors such as TGF-β and IL-6, thereby constructing a multi-level immune-escape network (12, 24). Among these, TGF-β has the capacity to inhibit the activities of immune cells including T cells and NK cells, while simultaneously promoting the differentiation of regulatory T cells (Tregs), thus giving rise to an immunosuppressive microenvironment (25). Moreover, VEGF can stimulate tumor angiogenesis, forging a vascular network that is conducive to the survival and metastasis of tumor cells, providing tumor cells with the means to evade the body’s immune surveillance (26–28).

This investigation has unearthed the existence of substantial NECTIN3-NECTIN2 and WNT7B-FZD1 signal interactions between epithelial cells with high PMS and fibroblasts, suggesting that CAFs might reshape the immunosuppressive niche through purine-metabolism reprogramming. Epithelial cells with high PMS receive an increased quantity of signals from fibroblasts and stellate cells and, concurrently, transmit more signals to fibroblasts, stellate cells, and T cells. One of the consequences of this metabolic reprogramming is to drive the formation of the “stiff-cancer” phenotype characteristic of PC. Tumor cells and CAFs synergistically promote ECM deposition through a “metabolic symbiosis” relationship. For example, the high-purine-metabolism region (Cluster6) undergoes co-activation with sphingolipid metabolism and taurine metabolism and may influence ECM remodeling by modulating the activity of PKM (29). In response to this, the spatial-transcriptome data obtained from this study have revealed that epithelial cells with high PMS are diffusely distributed within the tumor area, which exhibits a high degree of correlation with the stiff-cancer characteristics of PC, suggesting that abnormal purine metabolism may serve as a potential driver for stromal sclerosis. In terms of pathway activation, common signaling pathways such as the TNF signaling pathway and the HIF-1 signaling pathway exhibit marked differences between fibroblasts and stellate cells in different PMS groups, implying that these pathways, particularly the hypoxic environment, may exacerbate the fibrosis of PC and initiate a vicious cycle (30). The transcription factors CTNNB1 and LEF1 may be involved in regulating the interaction between these two groups of cells, signifying their latent importance in maintaining the balance of cell-cell interactions and the homeostasis of purine metabolism. Within the intricate tumor microenvironment, when perturbations arise in cell-cell interactions and purine metabolic processes, the expression profiles and functional activities of CTNNB1 and LEF1 are prone to undergo alterations. Subsequently, through their reciprocal interplay and the orchestrated regulation of downstream target genes, these two transcription factors strive to reinstate the homeostasis of both cell-cell interactions and purine metabolism.

Studies have demonstrated that the inhibition of the activities of certain key signaling pathways within fibroblasts can reduce the production of extracellular matrix and promote the infiltration of immune cells (25).As previously elaborated, patients in the high-PMS group are frequently accompanied by an immunosuppressive microenvironment, characterized by low immune-cell infiltration scores and immune-pathway scores, as well as high expression levels of the majority of immune checkpoints. This microenvironment inhibits the functions of immune cells, making it arduous for them to effectively attack tumor cells, thereby reducing the efficacy of immunotherapy. Nevertheless, this also offers novel perspectives for enhancing the effectiveness of immunotherapy. Through the optimized regulation of purine metabolism, it is anticipated to break through the immunosuppressive microenvironment, enhance the activity of immune cells, and thereby improve the sensitivity of immunotherapy (21). It is worth noting that with respect to the phenomenon that the overall survival (OS) of patients with high PMS and high tumor mutational burden (TMB) is relatively poor, it is possible that the immunosuppressive microenvironment generated by high PMS nullifies the potential immune-activation advantage conferred by high TMB. Although high TMB implies that tumor cells carry more gene mutations and are, in theory, more readily recognizable by the immune system, the immunosuppressive environment obstructs the effective attack of immune cells on tumor cells, enabling tumor cells to evade the body’s immune surveillance and clearance, ultimately leading to a dismal prognosis for patients (31–33).

GMPS (GMP synthase), the rate-limiting enzyme in *de novo* purine synthesis, has a high expression level associated with tumor-cell proliferation and chemoresistance. Recent research has revealed that GMPS can influence the activity of the RAS-MAPK signaling pathway by regulating the level of GTP, thereby facilitating the progression of KRAS-mutant tumors (34). NT5E (CD73), functioning as an ectonucleotidase, catalyzes the conversion of AMP to adenosine and represents a core molecule in tumor immunosuppression (35, 36). This study has determined that high GMPS expression is positively correlated with MDSCs infiltration, and NT5E+ cells are spatially co-located with CAFs, suggesting that the two entities shape the inhibitory microenvironment through “metabolic-immune” synergy. NT5E is more likely to act as an intermediate mediator in the purine-metabolic reprogramming of the pancreatic-cancer tumor microenvironment (TME) and the formation of an immunosuppressive environment. From the perspective of spatial distribution, NT5E is predominantly expressed on tumor cells, with fibroblasts interspersed among tumor cells. This distribution pattern gives rise to a typical “stiff-cancer” environment in PC, resulting in a scarcity of CD8 T cells and being conducive to the formation of an immunosuppressive environment. In terms of function, NT5E may be involved in purine-metabolism reprogramming, influencing the metabolic states of tumor cells and their surrounding cells, and thus regulating cell-cell communication and interaction (37). Significantly, in the verification experiment of the co-culture model system, knocking down NT5E can reduce the growth and invasion capabilities of tumor cells, yet when co-cultured with CAF, this downward trend is alleviated. This indicates that NT5E plays a vital role in the inter-cellular signal communication between tumor cells and CAF, promotes the malignant progression of PC, and thus emerges as a key intermediate mediator in the purine-metabolic reprogramming and the formation of an immunosuppressive environment.

In summary, this study has not only comprehensively analyzed the characteristics of purine metabolism in the pancreatic-cancer microenvironment but also delved deeply into the impacts of abnormal purine metabolism on cell differentiation, interaction, and the efficacy of immunotherapy, and identified key representative genes GMPS and NT5E, particularly clarifying the intermediate-mediating role of NT5E in the purine-metabolic reprogramming of the pancreatic-cancer TME and the formation of an immunosuppressive environment. However, this study still has certain limitations. The current small sample size may affect the generality of the conclusions. Additionally, in the future, it could integrate organoid models and spatial metabolomics to dynamically analyze the spatiotemporal evolution laws of the metabolic-immune network, thereby providing a novel paradigm for surmounting the treatment dilemma of PC.

## Data Availability

The original contributions presented in the study are included in the article/**Supplementary Material**. Further inquiries can be directed to the corresponding authors.

## Supplementary material

Supplementary Figure 1
**(A, B)** Immuno-correlation analysis of NT5E, controlling for immune factors and infiltration of immune cells. **(C-F)** Evaluation of the therapeutic efficacy of NT5E in common cell cohorts for tumor immunotherapy.
