# Supplementary figures and images for: Unveiling purine metabolism dysregulation orchestrated immunosuppression in advanced pancreatic cancer and concentrating on the central role of NT5E
Source: Front Immunol. 2025 Apr 1;16:1569088. doi: 10.3389/fimmu.2025.1569088 (PMC11996659; doi:10.3389/fimmu.2025.1569088)

A

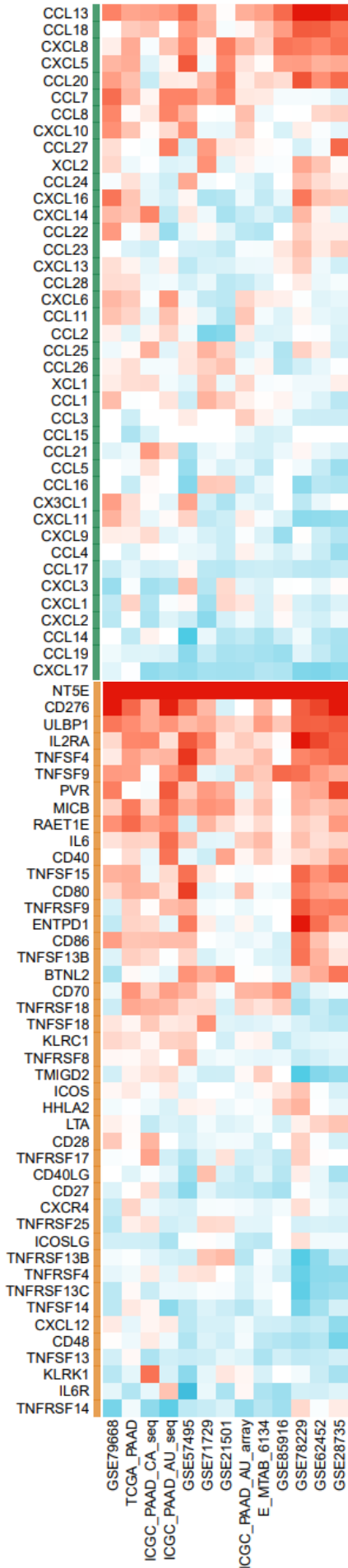

B

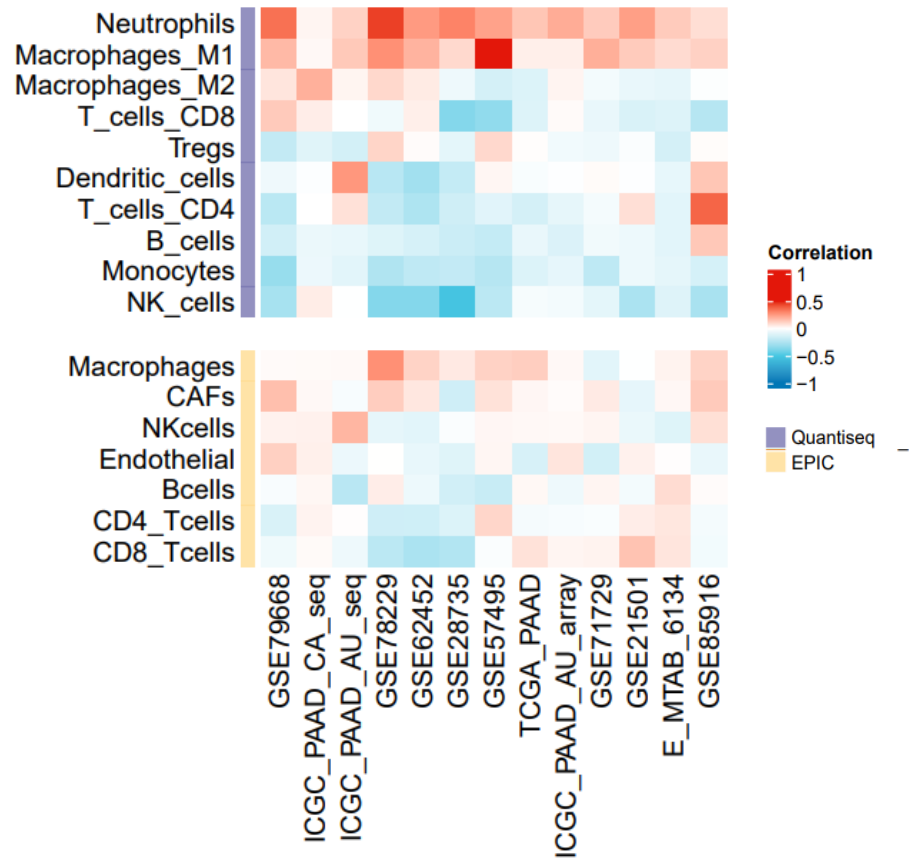

C

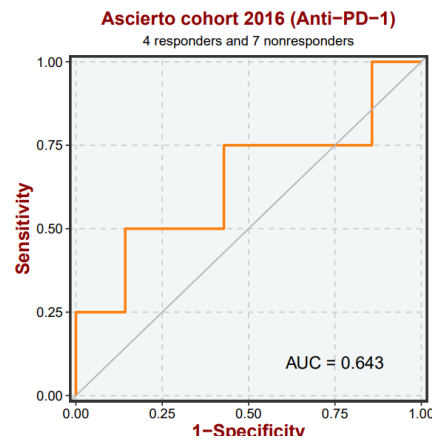

D

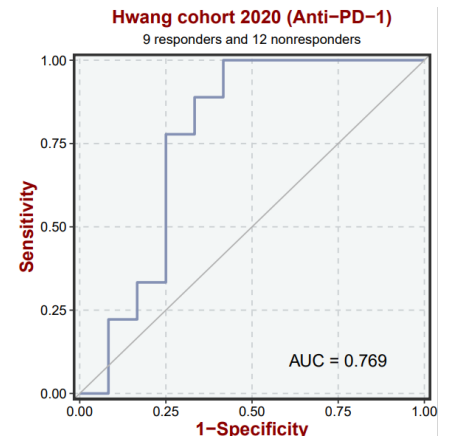

E

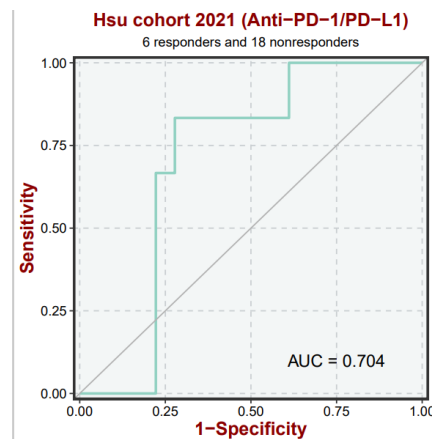

F

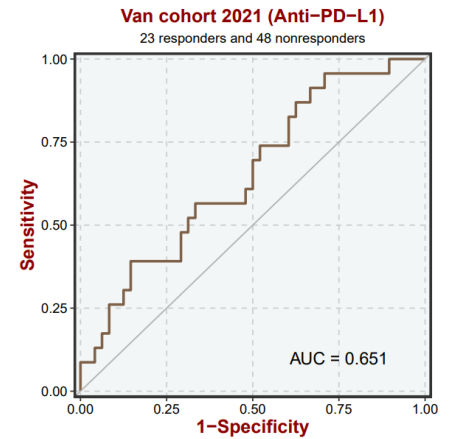

Supplement: Supplementary Figure 1 — (A, B) Immuno-correlation analysis of NT5E, controlling for immune factors and infiltration of immune cells. (C-F) Evaluation of the therapeutic efficacy of NT5E in common cell cohorts for tumor immunotherapy. [file Image1.pdf]
